# Commentary: Nuclear dynamics of the Set1C subunit Spp1 prepares meiotic recombination sites for break formation

**DOI:** 10.3389/fgene.2018.00496

**Published:** 2018-10-23

**Authors:** Csaba Fillér, Lilla Hornyák, Jason Roszik

**Affiliations:** ^1^MTA-DE Momentum Genome Architecture and Recombination Research Group, Department of Biochemistry and Molecular Biology, Faculty of Medicine, University of Debrecen, Debrecen, Hungary; ^2^Department of Genomic Medicine, The University of Texas MD Anderson Cancer Center, Houston, TX, United States

**Keywords:** meiotic recombination, double-strand break, histone methylation, Set1C, Spp1

An important element of the initiation of meiotic recombination is histone H3 lysine 4 trimethylation (H3K4me3) that marks meiotic DSB sites (Borde et al., 2009; Székvölgyi and Nicolas, 2010). H3K4me3 is deposited by the Set1C/COMPASS histone modification complex which contains seven subunits (Swd1, Swd2, Swd3, Bre2, Sdc1, Set1, and Shg1), of which Set1 is a histone methyltransferase (catalyzing the mono-, di-, and tri-methylation of histone H3 at lysine 4), and Spp1 regulates the tri-methylase activity of the enzyme (Nagy et al., 2002). In the current working model of meiotic recombination initiation, trimethylation of H3K4 is required for Spp1 to tether loop formation allowing DNA cleavage catalyzed by Spo11, a meiosis-specific nuclease (Keeney et al., 1997; Sommermeyer et al., 2013; Szekvolgyi et al., 2015). Spp1 has two important domains, a H3K4 binding PHD finger and a Mer2-interacting CXXC zinc finger domain, that are required for tethering the hotspot regions to the chromosome axis. Spp1 also plays a key role in trimethylation of H3K4 as it is able to bind the n-SET domain and interact with Set-c via the Swd3 subunit of Set1C/COMPASS to promote the methylation of H3K4 (Acquaviva et al., 2013a). In the absence of Spp1, H3K4me3 levels are greatly reduced (Acquaviva et al., 2013b). The chromatin structure generated through this process allows double-strand break (DSB) formation and cleavage.

A paper recently published by Karanyi et al (Karanyi et al., 2018) reports that the dynamics of chromatin binding of Spp1 plays an important role in meiotic DSB formation. In that article, the previous model of DSB formation, in which Spp1 is associated to Set1C and interacts with H3K4me3 and Mer2, is refined. In the new model the Spp1 and Mer2 interaction is independent of Set1C. The authors showed that Spp1 exhibits dynamic chromatin binding features during meiosis, dependent on the PHD finger and Mer2-interacting domains of Spp1 as well as on modifiable H3R2/K4 histone residues. The PHD domain may also contribute to the efficient chromatin binding of Spp1 at chromosome axis sites, but it is not a prerequisite for Spp1-Mer2 interaction. Detailed analysis of Spp1 chromatin binding suggests that the low turnover rate of newly appearing Spp1 binding sites leads to a stable and prolonged Spp1-Mer2 interaction, which may be a requirement for DSB formation. Adam et al. (2018) have also shown that Spp1 has distinct functions in the Set1C and DSB formation complexes in meiotic cells, and disrupting the Spp1-Set1 interaction does not prevent initiation of meiotic recombination. The Spp1-Mer2 interaction was found to be important for meiotic recombination initiation but not for histone H3K4 trimethylation mediated by the Set1 complex.

Ample cytological evidence supports the dynamic reorganization of meiotic chromosomes during the prophase of meiosis (Zickler and Kleckner, 2015; Muller et al., 2018), however, the tethered loop axis complex remains to be explored. In their pioneering study, Muller and colleagues designed and assembled a 144 kbp synthetic genomic region in *Saccharomyces cerevisiae* with an increased resolution for Hi-C experiments and tracked the behavior of this re-designed region during the first stages of meiotic prophase (Muller et al., 2018). Using this approach they were able to assess multiple fundamental features of spatial genome organization during meiosis, including chromatin restructuration into arrays of Rec8-delimited loops, chromosome individualization and pairing, and de-clusterings of centromeres. To identify direct spatial interactions of Spp1 and other proteins (e.g., Mer2, Hop1, Red1, and Rec8) that are required for loop-tethering during meiosis and elucidate the role and effect of Spp1 in DSB formation, high-resolution approaches like *in situ* Hi-C (Rao et al., 2014), Hi-ChIP (Mumbach et al., 2016), PLAC-seq (Fang et al., 2016), Capture Hi-C (Mifsud et al., 2015), Capture-C (Davies et al., 2016), or ChIA-PET (Tang et al., 2015) could be applied over the entire genome. Future spread of these C-based methods is expected to lead to a deeper understanding and exploration of meiotic chromatin structure in the context of the key role of Spp1.

## Author contributions

All authors listed have made a substantial, direct and intellectual contribution to the work, and approved it for publication.

### Conflict of interest statement

The authors declare that the research was conducted in the absence of any commercial or financial relationships that could be construed as a potential conflict of interest.
